# 2804. Azole versus echinocandin combination therapy for the treatment of invasive mucormycosis: A case series

**DOI:** 10.1093/ofid/ofad500.2415

**Published:** 2023-11-27

**Authors:** Meera Shah, Emily A Gibbons, G Christina Gutierrez, Kelly Reveles

**Affiliations:** University of the Incarnate Word Feik School of Pharmacy, San Antonio, Texas; University Health, San Antonio, Texas; University Health, San Antonio, Texas; The University of Texas at Austin, San Antonio, Texas

## Abstract

**Background:**

Mucormycosis is a rare and aggressive mold infection with an all-cause mortality of 54%. Treatment for mucormycosis has historically consisted of high dose liposomal amphotericin B (LAmB) as monotherapy. Recent data has emerged studying combination therapies; however, this data is conflicting, and the benefit of combination therapy remains unclear. This case series aims to explore different combination therapies utilized for the treatment of mucormycosis and their effect on mortality.

**Methods:**

We report 17 cases of proven or probable mucormycosis managed with combination therapy for at least 48 hours at University Hospital between March 2020 and November 2022.

**Results:**

The median age of patients was 60 years old (range 21-74), and 11 patients were male (64.7%), with a median BMI of 27.8 kg/m^2^. Of the 17 patients, all patients (100%) were Hispanic, nine patients (53%) had diabetes and two patients (12%) were lung transplant recipients. The most common infection site was rhino-orbital (6/17; 35%) and surgery was performed on 11 patients (64.7%) after a median of two days after hospital admission. All 17 patients received LAmB with an average initial dose of 5.13 mg/kg/day (range 2.9-6.4 mg/kg/day). All patients received combination therapy with either posaconazole (6/17; 35.2%), micafungin (4/17; 23.5%), or triple therapy (7/17; 41.2%). Duration of combination therapy was a median six days (range 2-21 days). Five of the 17 patients (29%) died of mucormycosis-related complications. Mortality observed in patients receiving combination therapy is as follows: micafungin combination (4/4; 100%), posaconazole combination (5/6; 83%), and triple therapy (0/7; 0%).

**Conclusion:**

Mucormycosis is an aggressive fungal infection with high morbidity and mortality that requires immediate recognition, urgent intervention, and appropriate antifungal dosing. Combination therapy with micafungin maybe associated with higher mortality compared to posaconazole or triple therapy. In this cohort, combination may be associated with lower mortality that previously documented but further investigation is required to determine optimal combination treatment for invasive mucormycosis.

**Disclosures:**

**Kelly Reveles, PharmD, PhD**, Ferring Pharmaceuticals: Advisor/Consultant|Ferring Pharmaceuticals: Honoraria

